# The prevalence of musculoskeletal pain and use of painkillers among adolescent male ice hockey players in Finland

**DOI:** 10.1080/21642850.2014.884463

**Published:** 2014-04-15

**Authors:** Harri Selanne, Tatiana V. Ryba, Kirsti Siekkinen, Heikki Kyröläinen, Hannu Kautiainen, Harto Hakonen, Marja Mikkelsson, Urho M. Kujala

**Affiliations:** ^a^ LIKES Research Center, Jyväskylä, Finland; ^b^ Institute of Sports Science and Clinical Biomechanics, University of Southern Denmark, Odense, Denmark; ^c^ Department of Biology of Physical Activity, University of Jyväskylä, Jyväskylä, Finland; ^d^ Med Care Oy, Äänekoski, Finland; ^e^ Päijät-Häme Social and Health Care Group, Lahti, Finland; ^f^ Department of Health Sciences, University of Jyväskylä, Jyväskylä, Finland

**Keywords:** musculoskeletal pain, painkillers, adolescent athletes, injury and prevention

## Abstract

Participating in competitive sport increases the risk for injuries and musculoskeletal pain among adolescent athletes. There is also evidence that the use of prescription drugs has increased among sport club athletes. The purpose of this study was to evaluate the use of painkillers among young male ice hockey players (IHP) in comparison to schoolboys (controls) and its relation to the prevalence of musculoskeletal pain and problems during activities and sleeping. Information was gathered through a questionnaire, completed by 121 IHP and compared to the responses of 618 age-matched controls. Results showed that monthly existing pain was at 82% for IHP, and 72% for controls, though IHP had statistically more musculoskeletal pain in their lower limbs (56% vs. 44%), lower back (54% vs. 35%), and buttocks (26% vs. 11%). There were no group differences in the neck, upper back, upper limb, or chest areas. The disability index was statistically similar for both groups, as musculoskeletal pain causing difficulties in daily activities and sleeping was reported by a minority of subjects. Despite this similarity, IHP used more painkillers than controls (18% vs. 10%). Further nuanced research is encouraged to compare athletes and non-athletes in relation to painkillers.

## Introduction

Sport participation is a highly valued social activity in Europe (European Commission, 2007). In Finland, for example, 24% of the population identify themselves as athletes and parents involve their children in organized sports at increasingly earlier ages (SLU, 2010). Despite numerous psychological, social, and health benefits of sport participation, researchers have also identified the increased risk for injuries and musculoskeletal pain among adolescent athletes (Auvinen, Tammelin, Taimela, Zitting, & Karppinen, 2008; Kujala, Taimela, & Viljanen, 1999).

Ice hockey is the most popular sport among male youth in Finland. During intensive training periods, the lower back, pelvic region and lower limbs of ice hockey players (IHP) are exposed to high loading. During matches, frequent contact at high speeds with each other and the boards (body checking) create a considerable risk for sustaining upper limb and upper body injuries and musculoskeletal pain (Mölsä, Kujala, Näsman, Lehtipuu, & Airaksinen, 2000; Sim, Simonet, Melton, & Lehn, 1987). While several reports have been published on injuries among players of different ages and divisions in ice hockey, little is known about the prevalence of musculoskeletal pain among adolescent male IHP (Benson & Meeuwisse, 2005; Emery & Meeuwisse, 2006; Kujala et al., 1995).

There is also limited data about the use of painkillers among adolescent players. Warner, Schnepf, Barrett, Donald, and Swigonski (2002) found that among adolescent high school football players, one out of seven players take non-steroidal anti-inflammatory drugs (NSAIDs) on a daily basis. Kelly and Parsons (2007) further reported on the increased use of prescription drugs among young athletes in sport clubs. Being key sites of social life among youth, sport clubs appear to endorse the use of painkillers rather than a break in training to treat musculoskeletal pain. However, the use of NSAIDs is beneficial only in soft tissue inflammatory pathologies, such as tenosynovitis and impingement, but not fractures, chronic tendinopathies or muscle injuries (Paoloni, Milne, Orchard, & Hamilton, 2009). The unhealthy influence of painkillers on gastrointestinal and kidney function as well as on cartilage is well documented (Kristensen et al., 2012; Waterman & Kapur, 2012) with a possibility of some rare but very dangerous side effects in liver functions (Lapeyre-Mestre et al., 2006). It has also been suggested that NSAIDs can cause malfunction of the muscle metabolism and, therefore, can be very harmful to young athletes' physical development (Mackey, 2007).

Consequently, the purpose of this study was to evaluate the prevalence of musculoskeletal pain and use of painkillers among adolescent, national-level, male IHP compared to age-matched schoolboys (controls). Further, we studied the influence of the pain on certain daily activities and sleeping disorders. The following research questions guided the study: (1) what is the prevalence of musculoskeletal pain among adolescent male IHP in Finland? (2) what is the effect of the pain on youth's everyday life? and (3) what is the IHP usage of painkillers compared to controls?

## Methods

### Participants

The ethics committee of the Central Finland Health Care District approved the study. Before the study, written consent was obtained from the parents of 121 young male national-level IHP in Finland (*M*_age_ = 15 years, *R*_age_ = 14–16 years). IHP answered a questionnaire (described below) before the beginning of a national ice hockey series. During this period, the players train intensively and participate in some practice matches. Their responses were compared to 618 age-matched schoolboys (controls), who had answered the same questionnaire. The mean age, age distribution and response rate (93%) was similar in both groups.

### Questionnaire

The questionnaire included items on the prevalence of pain in the neck, upper and lower extremities, upper and lower back, buttocks and chest area that had occurred at least once a month during the previous three months. Similar scales have been used in the research of Mikkelsson, Salminen, and Kautiainen (1996, 1997). Questions were also asked on the use of pain killers to treat the reported pain, and the respondents' estimates of the effects of this pain on various daily activities, such as walking more than one kilometer, sitting during lessons at school, participating in school physical education, engaging in hobbies, and also on ability to fall asleep or sleeping.

A disability sum index, as described by Mikkelsson et al. (1997), was calculated from the answers to the following statements: (1) I have difficulty in falling asleep because of pain and aches and/or pain and aches interfere with my sleep; (2) I have difficulty in sitting during a lesson; (3) pain bothers me if I walk more than one kilometer; (4) pain bothers me during physical education class; and (5) pain and aches interfere with my hobbies.

### Statistical analysis

Results were expressed as means with standard deviation or frequency counts with percentage counts. Inter-group statistical comparisons were performed by chi-square test or a permutation test – as appropriate. The *α* level was set at 0.05 for all tests.

## Results

**Figure 1.**
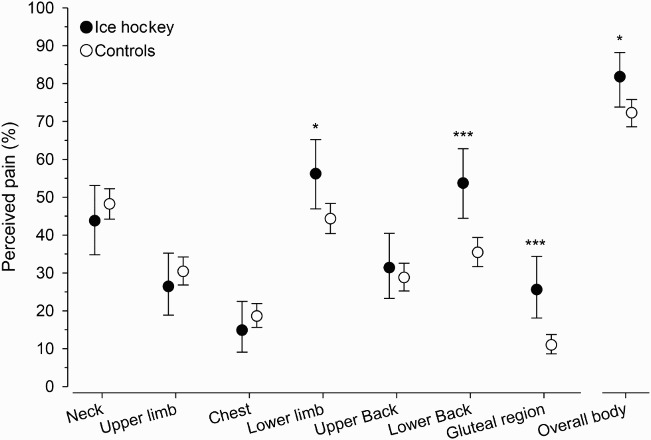
Perceived pain in musculoskeletal system in IHP and controls (*p < .05, **p < .01, ***p < .001).

Musculoskeletal pain occurring at least once a month was reported by 82% of the IHP compared to 72% of controls (*p* = .030). The prevalence of pain in the lower limbs (56% vs. 44%; *p* = .017), low back (54% vs. 35%; *p *< .001) and buttocks (26% vs. 11%; *p *< .001) was higher among IHP than controls. There were no statistically significant differences in the prevalence of pain in the neck (44% vs. 48%; *p* = .37), upper back (31% vs. 29%; *p* = .57), upper limbs (26% vs. 28%; *p* = .02) or chest areas (15% vs. 18%; *p* = .33) between the study groups (Figure 1). Only a minority of the respondents in both groups reported difficulties in daily activities and sleeping caused by musculoskeletal pain. These pain-induced problems were formed into a disability index, which was statistically similar for both groups (IHP vs. controls; 0.40 ± 0.92 vs. 0.47 ± 0.93; *p* = .43 by permutation test). The composite measures of the disability index are shown in Table 1. The results obtained further revealed that the use of painkillers by IHP was significantly higher than controls (18% vs. 10%; *p* = .008).

**Table 1.** Absolute and relative distribution of different daily activities and sleeping disorders among IHP and controls.

Disability index items: difficulties caused by musculoskeletal pain	Ice hockey *N* (%)	Controls *N* (%)	*P*-value
Falling asleep	4 (3)	33 (5)	0.35
Sleeping disorders	2 (2)	23 (4)	0.25
Sitting during school lessons	12 (10)	56 (9)	0.77
Walking more than 1 km	6 (5)	51 (8)	0.21
Participating in school physical education	13 (11)	62 (10)	0.81
Fulfilling free-time hobbies	11 (9)	68 (11)	0.53

## Discussion

The present study's results suggest that male adolescent IHP in Finland experience musculoskeletal pain at least once a month during an intensive training pre-competition period. Compared to the age-matched controls, IHP had more overall musculoskeletal pain, especially in their lower limbs, low back, and buttocks areas. There were no significant differences in the neck, upper extremities, upper back or chest pain between these study groups. The disability index caused by musculoskeletal pain was similar in both study groups, but regardless of that IHP used considerably more painkillers than their age-matched controls.

### Musculoskeletal pain

The prevalence of musculoskeletal pain among adolescents varies in previous studies according to the age of subjects, pain location and the time period used. To our knowledge, there are no studies about male adolescent IHPs’ musculoskeletal pain. According to the present study, the IHP seem to have more overall musculoskeletal pain than the controls, which is an expected result considering the nature of ice hockey as a contact collision sport with high speed and high impacts.

In previous studies it has been shown that low back and neck areas are the most common anatomical location of musculoskeletal pain in the adolescent population (Hakala, Rimpelä, Salminen, Virtanen, & Rimpelä, 2002; Kujala, Taimela, Erkintalo, Salminen, & Kaprio, 1996). When comparing “sports group” with “no sports group”, Sato et al. (2011) have indicated that the life-time prevalence of low back pain was significantly higher in the “sports group” population of Japanese children and adolescents. It has been further acknowledged that vigorous participation in physical activities is related to the self-reported low back pain (Auvinen et al., 2008; Kujala et al., 1999). Another study of 4667 Japanese students reported that excessive exposure to competitive sports activities during youth is associated with low back pain, and also symptoms in the lower extremities varied in different sport disciplines (Hangai et al., 2010).

In the present study, IHP reported more pain in the lower limb, low back, and buttocks areas than the controls, with a significantly higher percentage of monthly existing low back pain (54%).

In addition to the increase in low back pain from participation in competitive athletics, the demands of a specific sport affect the musculoskeletal pain experienced by an adolescent. In ice hockey, maintaining skating position loads the low back, and may therefore cause low back pain. Furthermore, at the age of 14–16 (this study's participants), training frequency and intensity increase while the musculoskeletal system of male players is still not fully developed. All these factors can lead to increased loading of the trunk and lower limb areas.

It is interesting that in this study, IHP did not have more musculoskeletal pain in their chest, upper back, upper limb or neck area than controls. This coheres with the findings of Feldman, Shrier, Rossignol, and Lucien (2002) who concluded that, although neck and upper limb pain is common among teenagers, sport involvement does not create a risk factor for the development of this type of pain. The fact that our questionnaire was administrated before the competition period might have contributed to the low prevalence indices of musculoskeletal pain in the upper body and upper limbs among IHP compared to controls. The heavy training during summer causes extra loading, especially to lower anatomical areas of the body, while the vigorous playing and cross checking during games tend to affect more upper body areas. Moreover, regular strength and conditioning training in ice hockey might have protected, to some extent, the ice hockey respondents from upper body pain and ache, because strength training has been established to be a preventive measure from neck and shoulder pain (Ylinen, Häkkinen, Nykänen, Kautiainen, & Takala, 2007).

### The use of painkillers and problems in daily activities caused by pain

Results obtained in this study suggest that musculoskeletal pain disturbed slightly such daily activities as walking more than one kilometer, sitting during school lessons, participating in school physical education, fulfilling leisure time hobbies, falling asleep or sleeping in both study groups. There was no statistical difference in these problems between the groups. Nevertheless the amount of painkillers used by IHP was twice as common as among the controls. There may be some benefits in treating inflammatory processes of the body with painkillers, especially with NSAIDs, but there may also be side effects.

According to Kelly and Parson's (2007) report of the alarming increase in the usage of prescription drugs among young sport club athletes, there is a danger that painkillers are distributed to young athletes by coaching staff to prevent potential pain (and not for real need) leading to misuse of painkillers in a sport club setting. Furthermore, dominant narratives in the ice hockey culture, such as “only real men play hockey” and “no pain, no gain”, contribute to normalizing pain as necessary in athletic pursuits. Thus, adolescent hockey players become prone to taking painkillers in order to train and participate in matches instead of taking a break due to musculoskeletal pain or injury. Such desensitization to painkillers that enable young athletes to train and perform better might be implicated in the developing of a “doping mentality” (Melzer, Elbe, & Brand, 2010), becoming a gateway to the use of forbidden performance enhancement substances.

Regular use of painkillers is known to cause undesired effects on stomach, liver and kidney functions, and on the metabolism in cartilage tissue (Kristensen et al., 2012; Lapeyre-Mestre et al., 2006; Redéen, Petersson, Kechagias, Mårdh, & Borch, 2010; Waterman & Kapur, 2012). Bodensen, Mill, Kegley, and Pavlath (2004) have further identified that NSAIDs have a negative influence on skeletal muscle myogenesis through cyclo-oxygenase pathway. Therefore, coaching staff and parents should exercise reasonable caution when administering painkillers to young athletes and instead educate athletes about healthier means of treating musculoskeletal pain, such as cold packs and rest from training to facilitate the recovery. Further studies are needed to investigate exact indications for the use of painkillers among adolescent athletes.

## Limitations of the study

In this research, respondents answered questions about musculoskeletal pain and use of painkillers retrospectively. Recall bias is a known limitation of retrospective questionnaire studies. In addition, ice hockey is a high collision sport, exposing the athletes to regular contacts and contusions, which may have contributed to IHP's divergent interpretations of pain than controls. At the age of the present study groups (14–16 years), some of the participants might still have growth pain, influencing the results obtained. Due to the fact that only male players participated in the research, conclusions concerning the effect of gender on the results cannot be made.

## Conclusion

Though musculoskeletal pain is more common in competitive adolescent IHP than in their peers, there is no significant difference in the neck, upper back, upper limb, or chest area pain reported by both groups. However, the adolescent IHP do demonstrate a higher percentage of musculoskeletal pain in the areas of the low back, buttocks, and lower limbs than age-matched controls.

In both groups, a minority reported some difficulty in performing daily activities and a disturbance in sleeping as a result of musculoskeletal pain. The intensity of this pain is not high, but in contrast with the controls, IHP report using a higher percentage of painkillers to diminish these effects. This raises the question of whether IHP are receiving the appropriate dosage of painkillers, corresponding to their level of musculoskeletal pain, and, indeed, what is the best way to treat this pain according to good clinical practice.

Further nuanced research is necessary to determine whether the similar levels of musculoskeletal pain reported by IHP and controls is as a result of or despite the disparate usage of painkillers. There is further need to establish whether adolescent athletes are being given and are taking prescription painkillers in a manner appropriate to their injuries and reported pain, or whether the disparity between painkiller usage between IHP and controls reveals a misuse of these resources. Further studies could also investigate the effect of gender on self-reported pain and usage of painkillers in ice hockey and other sports.
